# Supplementary material for: Are carbon emissions trading and green financial instruments synergistic? -Comprehensive quantitative research based on content analysis
Source: PLoS One. 2024 Mar 7;19(3):e0298601. doi: 10.1371/journal.pone.0298601 (PMC10919601; doi:10.1371/journal.pone.0298601)
Supplement: S1 Data — (ZIP) [file pone.0298601.s001.zip › Data/地级市Z_F工作报告.zip!/╔╜╬≈/┤≤═1⁄4╩╨/└┤╫╘╣1⁄2╓┌║┼í╛┬φ┐╦╩2╛▌═°í┐_2018.docx]

各位代表:

现在,我代表市人民政府,向大会报告工作,请予审议,并请市政协委员和其他列席人员提出意见。

一、2017年工作回顾

过去的一年,全市上下高举习近平新时代中国特色社会主义思想旗帜,认真贯彻党的十九大精神和新发展理念,全面落实省委“一个指引、两手硬”重大思路和要求,大力实施“136”发展战略,经济建设和各项社会事业取得可喜成绩。

——经济增长回归合理区间。地区生产总值完成1121.8亿元、增长6.5%,在全省排名由末位上升到第7。规模工业增加值完成285.9亿元、增长5.7%,排名由全省末位上升到第7。固定资产投资完成489.3亿元、增长7.0%,社会消费品零售总额完成654.5亿元、增长7.5%,两项指标增速均列全省第1。公共财政预算收入完成108.3亿元、增长21.8%,全省排名第4。城乡常住居民人均可支配收入分别达到27981元和8862元,分别增长6.5%和7.9%,分别排全省第5和第2。

——转型发展态势强劲。以非煤产业为主攻方向,聚焦新兴工业和高科技工业,大力发展实体经济,新增规模企业66家,41家为战略性新兴企业,全年非煤产业增加值完成157.1亿元,同比增长14.5%,占全市规模工业增加值比重达到54.9%,拉动工业经济增长9个百分点。下大气力推进经济转型发展和产业重点转移,战略性新兴产业增加值完成27.8亿元,同比增长18.4%,占比提高9.7个百分点。高技术产业增加值完成19.5亿元,同比增长1.3%,占比提高6.8个百分点。2017年风力发电、光伏发电装机规模分别占到全省的30%和40%,新能源装备制造初具规模。下大气力推进煤炭清洁高效利用,全市煤炭清洁高效利用率同比增长6.8%,煤炭清洁高效转化率同比增长13.9%,5000吨玄武岩纤维项目、2万吨石墨电极项目正式投产,20万条高铁受电弓项目正式进入试验阶段。成功引进北京汉能、深圳科陆、雄韬股份、香港正道等大型清洁能源集团,签约落地新能源、新材料、新技术等转型标杆项目28个,总投资416.8亿元。制定出台落实国发〔2017〕42号文件的实施意见,转型政策体系基本确立,主动转型、创新转型、深度转型、全面转型的氛围初步形成。

——发展动能接续转换。供给侧结构性改革继续深化,淘汰煤炭落后产能415万吨,煤炭产业占全市规模工业的比重同比下降3.6个百分点。争取地方新增债券、置换债券67.6亿元。第三产业增加值完成646.1亿元、增长7.4%,占比达到57.6%,高于第二产业20.8个百分点,对GDP增长贡献率为57.1%。新登记市场主体增长20%,达到3.3万户,主要聚集在第三产业。旅游业总收入完成483.1亿元、增长33%,增速全省第一。大同被确定为全省唯一的国家级旅游业改革创新先行区。创新驱动成效显著,中科院、北大、清华等高端智库入驻大同,大数据产业、新一代人工智能信息、康养、医疗、“互联网+”等新业态全面推进。

——对外开放步伐加快。与省教育厅签订了大同大学市厅共建协议,率先开启市厅共建大学模式。与省外侨办签署市厅对外开放发展工作协议,有力促进我市对外开放和交流合作。与省电力公司签署合作协议,为我市创新驱动、转型升级提供优质电力保障。与中科院7个所签订新能源发展共建合作协议,聘请中科院院士李灿为大同新能源产业战略咨询委员会主任。中科院全科盟在大同注册成立了大同新能源产业技术研究院,为煤炭清洁高效利用建立了研发平台。与太原铁路局集团公司开展深度合作,“云冈号”直通旅游专列有效促进大同与太原的旅游发展。云冈机场旅客吞吐量达64万人次、增长17.55%。大同国际陆港进口肉类指定查验场通过审核验收。对接“一带一路”,法国文化名人阿兰·蓬皮杜就大同建设法国蓬皮杜国际艺术社区进行了考察,并积极推动建设大同国际艺术社区项目。加强大气污染防治合作,积极促成韩国环境部长金恩京来同考察,召开了韩国优秀技术说明会。中博会、“丝路行”等招商引资活动成功签约重大项目42个、总签约资金额700余亿元。

——城乡建设统筹推进。完成道路工程82.2公里,新建和改造城市供热管网152公里、供气管网224公里、供水管网138公里、污水管网71.5公里。古城保护与修复步伐加快,代王府、潘家园、晋商联盟等项目全面推进,永泰门广场立体通道于11月18日全面通车。御东新区功能不断完善,国际会展中心开工建设,体育中心、大剧院、美术馆后续工程有序推进。智慧城市加快创建,数字化管理平台基本建成。高铁枢纽项目启动实施。大张高铁加快建设,大乌高铁前期进展顺利,大西高铁有序推进,大县城建设成效显著,城乡面貌焕然一新。

——生态环境持续改善。突出建管并重、铁腕治污,加强禁煤区建设,推进煤改气、煤改电工程,完成煤改集中供热5.9万户、300多万平方米,淘汰燃煤锅炉243台,取缔“散乱污”企业610家,全市二级以上优良天数304天,全省排名第1。全面落实河长制,治污水、防洪水、排涝水、保供水、抓节水,“五水共治”工程扎实推进。桑干河治理工程开工建设,十里河入御河生态湿地工程全面加快,全市10条主要河流12个考核断面Ⅲ类及以上优良水体比例、劣Ⅴ类断面的比例均达16.67%,水环境质量稳步好转。全年完成造林34.5万亩,森林覆盖率达到23.5%。城市建成区绿地率达到37.7%,人均公共绿地面积15.4平方米,成功入列2017美丽山水城市。

——精准脱贫成效明显。实施脱贫攻坚“八大工程二十项行动”,148个贫困村摘帽、7.2万贫困人口脱贫。扶持贫困村发展小杂粮、中药材、黄花等特色产业,带动2.82万人脱贫。建成光伏扶贫电站13座,惠及321个贫困村。灵丘车河、城头会等4个旅游脱贫村成为全省典型。建成易地扶贫搬迁安置点93个。组建造林专业合作社263家,带动1.14万人脱贫。“天镇保姆”等劳务品牌效应凸显,就业扶贫渠道不断拓宽。社保体系进一步完善,政策托底成效明显。汇集社会扶贫资金5000万元,85家企业认领扶贫项目350个,“扶贫超市”的暖心举措让老百姓得到实惠。

——民生福祉不断提升。坚持以人民为中心的发展思想,财政用于民生事业支出266亿元,占总支出的81.7%。义务教育标准化建设不断加快,完成“全面改薄”年度任务,义务教育均衡发展基本实现全覆盖。城镇新增就业4.48万人,农村劳动力转移就业2.39万人,城镇失业登记率3.1%,低于控制目标。市级公立医院全部取消药品加成,全省首家进行药品二次议价,实现药品费用和药占比双下降。在全省率先实现“三保合一”,覆盖全民的医保和养老保险体系基本建成。完成老旧小区改造1.3万套,棚户区改造建成9万套,新增集中供热面积450万平方米。11件民生实事全部兑现。行政综合执法体制改革走在全省前列。社会治理、安全生产、信访维稳等工作扎实有效,人民群众的获得感、幸福感、安全感显著增强。

——干事创业劲头更足。坚持作风建设永远在路上,政府系统政治建设全面加强,广大干部在“两学一做”学习教育、贯彻落实党的十九大精神和习近平新时代中国特色社会主义思想中得到锤炼,焕发出强烈的干事创业热情。市住建委5天建成中银纺织项目2350米施工道路,供电公司6天架设3.4公里电力线路,大同县10天打成188米深井。市工商局1个半小时为中科院全科盟新能源产业技术研究院办理工商注册手续。市政府与同煤建立常态化例会工作机制,5次深入集团对接项目、共谋发展,首次例会为同煤集团解决13个问题。开发区、发改、经信、教育、住建、规划、商务、市政、城投、外事等多个部门的广大干部加班加点定任务、赶进度,促进了今年各项工作的早部署、早安排,大家倍加勤奋工作的劲头和释放出的大力度、快节奏、高效率的正能量,正在逐步成为全市广大干部的自觉行动。大家争先进位、勇争第一的工作标准,已经逐步形成广大干部自觉信守的思想共识。我们要倍加珍惜这种正能量,把这种正能量凝聚成一股强大的“文化磁场”,辐射人人,激增倍长,让每一个干部、每一份事业都连点成线,以线带面,着眼正道,全力拼搏,快步而行,决不辜负全市人民群众的信任和期望。

各位代表,上述成绩的取得,根本在于习近平新时代中国特色社会主义思想的科学指引,是省委省政府正确领导的结果,是市委科学决策、市人大有力监督、市政协大力支持、全市上下苦干实干的结果。在此,我代表市人民政府,向全市人民,向各民主党派、工商联和各人民团体,向全体人大代表、政协委员,向驻同部队、公安民警和各级驻同单位,向离退休老同志,向所有关心支持大同改革发展的各界朋友,致以崇高的敬意和衷心的感谢!

回顾过去,我们取得了一些成绩,但同时我们也清醒地认识到,经济社会发展中还面临许多深层次的矛盾和问题。一是全市经济总量偏小,综合实力与全国13个较大城市和全省第二大城市的地位不相匹配,高质量发展的任务和压力巨大;二是“一煤独大”等结构性体制性素质性矛盾解决的力度和办法还不多,经济增长方式的转变还仍在“爬坡过坎”阶段,新兴产业仍在加快培育,转型发展依然任重道远;三是民生保障能力相对不足,经济社会发展不平衡不充分现象仍然凸显,脱贫攻坚任务艰巨,生态环境面临严峻挑战,城乡协调发展和可持续发展的统筹力度还不够;四是改革的力度还不够彻底,全方位开放的局面尚未形成,外向型经济发展明显不足,创新发展亟待持续深入;五是一些干部慢作为、不作为问题不同程度存在,依法行政水平不高,一些部门的服务意识、落实能力、主动担当的劲头与发展的要求和人民期盼还不相适应,对此,我们一定要高度重视,下大气力认真加以解决。

二、2018年经济和社会发展主要任务

2018年是贯彻落实党的十九大精神的开局之年,是改革开放40周年,是决胜全面建成小康社会、实施“十三五”规划承上启下的关键一年,做好今年工作,意义重大,影响深远。

今年政府工作的总体要求是:全面贯彻党的十九大和十九届一中、二中、三中全会精神,坚持以习近平新时代中国特色社会主义思想为指引,坚定不移贯彻新发展理念,按照高质量发展要求,统筹推进“五位一体”总体布局和协调推进“四个全面”战略布局,坚持稳中求进工作总基调,坚决打好防范化解重大风险、精准脱贫、污染防治攻坚战,按照省委“一个指引、两手硬”重大思路和要求,紧紧把“供改”和“综改”贯穿经济工作始终,围绕示范区、排头兵、新高地三大目标,深入推进“136”发展战略,打造先行区,当好两个尖兵,实施三大振兴,以“十大行动”推动质量变革、效率变革和动力变革,全面开创新时代大同发展新局面。

经济社会发展主要预期指标是:地区生产总值增长7%左右;第三产业增加值增长7.5%;规模工业增加值增长6.5%左右;固定资产投资增长10%;社会消费品零售总额增长7%;公共财政预算收入增长7%左右;城乡常住居民人均可支配收入分别增长6.5%左右和7%;对外贸易进出口总额增长5%;居民消费价格涨幅控制在3%左右。各项约束性指标和转型考核指标完成省下达任务。

做好今年工作,要坚决做到“三个必须”:

第一,必须坚定不移推进大转型、大开放、大创新。站在新的历史起点上,我们要把大转型作为破解资源型地区经济发展困境的唯一出路,克服“速度情结”和“换挡焦虑”,坚定不移推动经济发展从“速度经济”转向“质量经济”,(下转第二版)

(上接第一版)坚定不移推动发展从“有没有”实现“好不好”,坚定不移落实全省转型项目建设年重大部署,今年要以能源革命十大突破工程,引领全市转型发展项目落地生根,引领全市各项任务目标开工、成型、见效,形成转型发展强大合力。要把大开放作为加快高质量发展的必然选择,我们必须“跳出大同看大同,立足全省全国看大同”,要通过开放激发内生动力,借力“一带一路”增强外部合力,在激烈竞争中找准大同新的历史方位,推动形成互利共赢、全面开放新格局,打造新时代大同转型发展的“大脑”。要把大创新作为高质量发展的不竭动力,要聚焦煤炭清洁高效利用,推动技术升级,要聚焦“两个尖兵”和“工业振兴”,广泛吸纳人才,引深“品质革命”,打造大同高质量发展的升级版,推动大同质量变革、效率变革、动力变革走向新高地。

第二,必须坚持不懈对接大企大校大院大所。要强化四大对接意识。一是强化对接大企意识,主动走出去,对接世界500强、中国500强,引进更多的高端装备制造企业,加快大同经济结构调整,构建实体经济重要支撑。二是强化对接大校意识,主动对接清华、北大以及国际名校,向“双一流”看齐,加快培养一流人才、尖端人才,为转型发展培育第一资源,积累第一资本。三是强化对接大院意识,积极对接中科院、社科院,以市场化机制促进科研成果转化落地,把实验室的科研成果变成工厂车间的生产力。四是强化对接大所意识,盯住世界级前沿研究方向,加大科研经费投入,扶持建设院士工作站、科技研发中心,积极对接国家级、国际级高科技研究所,攻克关键技术,为实现“大同制造”向“大同创造”“大同智造”转变创造坚实基础。

第三,必须坚定不移坚持以人民为中心的发展思想。人民对美好生活的向往,就是我们的奋斗目标。我们所做的一切工作,都是为了人民。要把民生普惠作为高质量发展的出发点和落脚点,从我市实情出发,尽力而为、量力而行,把群众最关切最烦心的事一件一件解决好,促进社会公平正义和人的全面发展,使人民生活随着大同的发展一年更比一年好。要尊重群众的首创精神,坚持力量源于人民、奋斗属于人民的人民情怀,最大限度地激发广大干部群众的积极性、主动性和创造力,形成众人划桨开大船的生动局面。要把人民拥护不拥护、赞成不赞成、高兴不高兴、答应不答应作为衡量一切工作得失的根本标准,着重从老百姓不满意的一点一滴改起,努力建设人民满意的政府。

围绕总体要求、“三大必须”理念,今年要重点实施好十大行动:

(一)能源革命突破行动

能源革命突破行动,是资源型地区走出转型升级发展新路的一个重大举措。我们要以能源革命为突破,有效解决并逐步摆脱“煤炭依赖”,着力构建现代能源经济体系,推动大同新能源装备制造破冰和新能源供给提速,实现能源高端制造能力、非化石能源装机规模和消费水平在全省领跑,为推动“煤都”向“新能源之都”的转变,搭建一个看得见、能引领的“路径”和“样板”,为资源型地区走出新路提供大同模式和方案。今年要重点抓好十大项目。一是大同大昶移动能源有限公司铜铟镓硒薄膜电池项目,建设年产300兆瓦铜铟镓硒薄膜电池生产线,确保达产达效,填补大同新能源装备制造业技术的空白,实现新能源装备制造业零的突破。二是山西米亚索乐装备集成科技有限公司铜铟镓硒薄膜电池生产线核心设备及核心材料开发、制造、组装项目,主要引进国外核心装备建设薄膜电池4.0制造中心和靶材中心。三是西安隆基集团公司500兆瓦单晶硅太阳能电池组件项目,建设年产500兆瓦超高效率组件生产线,年底投产。四是雄韬股份30万套氢燃料汽车总成项目,形成30万套氢燃料汽车总成能力。建成2座加氢站,开通50辆氢燃料公交试营线,销售5万套氢燃料汽车总成。五是深圳科陆公司年产1000兆瓦时储能系统项目,主要生产储能系统、储能电池包PACK系统、BMS电池管理系统等。六是大同高迈北宇新能源汽车有限公司年产10万辆新能源电动重卡项目,建设1000辆整车装配线。年内形成装配1000台整车的动力和动力电池生产能力。七是大同经济技术开发区多能互补集成优化国家示范项目,总装机容量393兆瓦,年供电量为12.5亿千瓦时,今年一期建成投产。项目的建成对节能降耗和碳减排方面具有突出贡献,对单位地区生产总值能耗和二氧化碳排放两个指标,有直接的减排降耗贡献,将为全省智慧能源作出示范。八是国家光伏应用示范基地总装机容量50万千瓦光伏发电项目,今年年底建成并网,实现大同国际陆港和医药园区冷热电智能化三联供。到年底,全市新能源供给将进一步提速,装机总量可达545万千瓦,占全市电力总装机的36.5%。全部并网后,新能源工业增加值约75亿元。九是同煤集团同忻煤矿1000万吨智能化开采项目,该项目建成后,将成为全市深化能源供给革命、提高传统能源能效,实现煤炭走“减”“优”“绿”发展之路的样板,推动煤炭清洁化、绿色化程度大幅提升。十是建设大同市国际能源革命科技创新产业园,占地1000亩,“科创园”工程突出搭建研发平台、孵化平台、成果转化平台,围绕三大平台功能,全力实施“11+1”项目,即中科院清洁能源转化大同基地、全科盟大同新能源产业技术研究院、同煤“双创”中心、汉能北方新能源研发中心、“氢都”之星总部基地、协鑫未来能源馆、清华启迪新能源科技城、上海田鼎新能源与金融科创城、中以国际能源科技成果合作中心、网宿新能源大数据中心、泰瑞新能源新材料研发中心的11个项目,“+1”是在开发区同期开工建设中科院工程热物理研究所大同分所,建设总规模102.7万平方米。十大能源突破工程年内主体工程全部竣工,今年投资178亿元,当年可实现产值约80亿元。这十大工程是当好全省能源革命排头兵之尖兵的标杆项目,是推动大同能源革命的风向标,这十大工程的主体企业要挂图作战,倒排工期,列出投资进度,抓工期、抓进度、抓质量,确保按期开工、按期建成、按期投产。我们现已成立了书记、市长、同煤集团董事长、总经理、大同大学书记、校长任组长的能源革命领导组,所有副市长、副总经理、副校长都任副组长,并设立1亿元的科研奖励资金、10亿元财政扶持资金,组织100亿元规模的产业资金,搭建了政府、科研院校、企业为一体的实施班子,在全市上下已形成了一定要当好“尖兵”的浓厚氛围,一个全新的“氢都”“能谷”“智谷”“新能源产业之都”的蓝图,将变成今年实实在在能源革命的现实。

(二)工业振兴行动

大同是山西能源重化工基地的典型,是全省的老工业基地,有着工业发展的雄厚基础,从2017年规模工业增加值占全省比重仅为5.8%的贡献数据足可看出,工业振兴的重要意义和工业振兴的刻不容缓。今年我们的主要目标,就是要牢固树立工业立市、工业富市、工业强市的发展理念,举全市之力大抓工业、强抓工业,以工业振兴提质增效,重铸大同第二大工业城市的辉煌,今年工业振兴行动要突出抓好四大重点工作。一是突出抓监测。成立以市长任组长的工业经济运行服务组,从经信、煤炭、财政、国税、地税、统计6个部门抽调10名干部,专门腾出办公室,对工业经济运行进行“六个一”监测服务,即每月用一旬时间到企业监测服务,了解企业运行中存在困难和问题;每月清零一批企业反映的问题和事项;每季度召开一次工业经济运行分析调度会议;每半年对工业经济运行目标进行一次评估;每半年对部门支持工业转型进行一次考核,并纳入对部门的年度考核;全年对工业经济运行目标完成进行一次奖励兑现,确保制约企业运营效益的各类因素全部消除,实现无障碍发展。二是突出抓奖励。市财政列入5亿元,从转型速度、科技高度、技改力度、投资强度、建设进度等10个方面,对今年开工项目和技改项目进行大奖励、硬扶持,充分调动规上企业的技改积极性,通过技术挖潜增强内生动力,提高生产力。三是突出抓现有规模企业提质增效。从100个企业中,筛选高科技企业30户、新兴战略型企业30户、潜力增长企业40户,分三大方阵进行扶持培育。这100个项目,包括新建项目65个,技改项目35个,总投资1273.6亿元,全部建成投产后,预计实现销售收入1220.5亿元。主要涉及同煤集团、中车大同机车、大齿集团、陕汽集团、同药集团等十大企业,以及以同煤集团塔山矿、同忻矿为主的十座转型试点煤矿。同时,推进煤矿减量重组,3座矿井进入联合试运转并力争验收投产,新增先进产能240万吨。电力行业,重点抓好大唐国际云冈热电、二电厂、漳电大唐塔山3户企业的7个燃煤机组节能改造项目。这些项目,是事关大同工业转型发展的重大项目,必须全力扶持,全面推进。四是突出抓高科技工业和新兴工业。目前已确定“好项目”“大项目”176个,其中战略性新兴产业项目109个,从3月1日至今为新开工项目手续实行了集中办理,为4月16日集中统一开工打下了良好的开工基础。上半年,开工的龙头项目有,中广核风力发电制造总装项目、中美传奇轻型飞机制造项目、北京迪默透轻型飞机发动机项目、山西宁宝新能源科技公司石墨烯项目、高品质蓝宝石半导体晶体制造项目、现代化纺织智能制造项目等十大项目。我们的目标是,既要加快新产品、新工艺、新技术的开发引进和成果转化,又要引进高端装备制造、高科技环保节能、煤炭清洁高效利用、精密医疗设备、生物及生物制药工程、信息技术等重大产业项目,为构建多元发展的现代工业体系提供重要支撑。

开发区和各县区要把项目建设作为经济工作的生命线,结合各自实际,确立转型项目,狠抓转型项目的谋划,狠抓转型项目的引进,狠抓转型项目的建设,形成你追我赶、时不我待的转型劲头,争当壮大工业经济的排头兵。全市企业和企业家要积极围绕市场研究项目、围绕产业谋划项目、围绕发展推进项目,不断增强企业核心竞争力。要以工业振兴的提质增效,搭起大同重振工业雄风的新骨架;要以工业振兴行动的效益,迅速转变为大同经济版图新的增长点和增长极。

(三)对外开放行动

对外开放是提升大同外向型经济水平、增强综合实力的关键之举。要坚持走出去与请进来相结合,大力实施对外开放新高地提升工程,加快建设“制度高地”“环境高地”“创新高地”,打造融入京津冀桥头堡。今年重点抓好六大工程。一是强化与商务部投促局常态化对接工作机制。落实战略合作协议,借船出海,招大引强,依托大同经济技术开发区创建承接京津冀、长三角、珠三角产业转移国家级示范园,着力打造大同国家级东部产业转移示范区,年内力争引进东部产业转移重大牵引性带动性强的10大引领项目。二是建立赴京津冀和上海漕河泾开发区挂职锻炼常态化工作机制。目前已有16名干部挂职到位,4月10日前再增派10名干部挂职到岗。挂职的核心任务是,通过挂职学理念、学模式,达到引进项目、推进转型的目的。市直部门和各县区要积极参加和开拓多渠道进行挂职招商。三是市政府在北京、上海、深圳、雄安新区、以色列特拉维夫分别设立京津冀、长三角、珠三角、雄安新区、以色列高科技产业转移项目处,目的就是打造出一个全面开放的产业转型共享平台。目前中以经济合作大同产业园和希伯莱大学技术转移中心项目已挂牌。要加强与“一带一路”沿线国家经贸往来,实现年内出口贸易额增幅达到10%以上,与蒙俄贸易增幅达到20%以上。四是扩大经贸合作。以中柬建交60周年为契机,与柬埔寨暹粒市签约发展友好城市,签订了每年进口20万吨大米的农产品发展项目,增设通往金边和暹粒市等重点城市的直飞航班,目前大同直飞柬埔寨航班达到55班次、近两万人次,今后将在教育、文化、旅游、能源等7大领域与柬埔寨展开深度交流合作,特别是要用好文化旅游这张名片,让云冈石窟和柬埔寨的吴哥窟两大世界性文化品牌交相辉映。加强与芬兰文化交流,与芬兰国内城市缔结友好城市,启动占地100亩、建筑面积8200平方米的大同中芬启迪冰雪体育中心工程,建设2个世界一流的滑冰场馆和中芬文化交流中心,开展冰雪运动交流合作,促进大同冰雪装备产业发展。承办好大同中芬冰雪体育文化节活动。五是提升口岸吞吐能力。今年要新增国内航线14条,直通北京、上海、厦门、哈尔滨、武汉、深圳等20个城市,联通华东、东北、华南、西北、西南等各大区域。同时开通更多国际航线。加快大同国际陆港10大功能区建设,建成现代化区域性、综合性国际陆港。加快晋北物流园项目建设,打造立足大同、服务山西、面向全国的重要枢纽物流中心。积极争取开通中欧班列,提高外向型经济含金量。六是强化中小学生人文交流。从今年起,每年组织1000名中小学师生利用寒暑假开展国内外研学交流,开阔眼界,增长见识,为大开放大发展从小学开始就奠定下良好的人才基础,培养大同具有国际视野的长远力量。

(四)城市提质行动

城市是市民生活工作的家园。今年城市提质行动重点是大力实施老城区、御东新区、同煤新区、开发区“四区”同城化发展战略。老城区是我们大同历史文化和古都文化的标识;御东新区是我们打造区域性中心城市的新的目标区域;同煤新区是我们全市新旧动能经济发展的聚集地;开发区是我们新经济、新业态发展要素聚集的主战场。“四区联动”就是要实行统一规划、统一标准、统一建设、统一管理的城市经营理念,把我们的城市建成生态宜居、魅力宜游、安居宜业的美丽城市。今年重点抓好四大工程。一是古城修复工程。今年全面启动开工古城内代王府、九龙壁、大同府衙等18项修复工程,延续古城文脉。开工建设21万平方米全省面积最大广场——永泰门广场工程,年内投入使用。建成全省最大的北魏辽金平城百座博物馆,守住大同文化的根。全面推动东小城项目开工启动。全面启动拥军路片区一桥六路、西花园片区城建工程,方便老城区群众出行,推动今年古城等项目年底全面收工。二是御东新区工程。御东体育中心、大剧院、美术馆三大场馆必须完工,国际会展中心加快建设,完善城市公共服务功能。开工建设高铁站广场,拉开高铁枢纽建设大幕。三是道路工程。启动全市第一条连接同煤集团的20公里快速干线,西起同煤集团东到云冈机场,形成主干道路与外环高速有机衔接的交通体系,实现“四区”10分钟快进快出、半小时直达各县区。开源桥10月1日全线通车,形成御东御西第六大通道。建成大同现代纺织新城“三横九纵”路网工程,提升开发区承载能力。四是绿化工程。全面推进御河西路、魏都大道等城市绿化工程,投资38.1亿元。开工建设御河北拓南延生态工程,投资7.2亿元,将御河两岸建成市民休闲健身、宜居宜游的好去处。要把提高城市管理水平作为推动高质量发展的重要举措、为市民创造高品质生活的必然要求,加速城市国际化、现代化、智能化步伐,提升开放能级,不断增强城市吸引力、创造力和竞争力,在全省“一核一圈三群”城镇化布局中展现独特魅力。要重视城市总规的引领和约束作用,统筹协同城市在区划设置、产业培育、文脉延续、市民安居、生活服务等多种功能,通过“四区联动”实现整体提升;加快推进创新城市、智慧城市、绿色城市、海绵城市、人文城市建设,完善城市空间、道路及地下综合管廊等基础设施,全方位、大幅度提升城市承载力;要充分发挥市民参与城市建设与管理的能动性,聚集广大市民智慧,持续深化城市综合执法管理体制改革,从老百姓身边小事管起,打磨城市品质,涵养城市精神,讲好大同城市每一个角落的故事。

(五)乡村振兴行动

乡村振兴行动是新时代“三农”工作的总抓手,是全面建成小康的关键。今年,乡村振兴重中之重是抓好“一带三区”。一是建设雁门关农牧交错带。打造南郊、阳高、天镇、左云、新荣、浑源6个特色养殖大县。加快培育奶牛、生猪、草食畜3个产业集聚区。推进阳高正大、广灵海高、浑源正邦等农牧业重大项目建设。创建大同黄花、广灵食用菌省级现代农业产业园和左云县沙棘特色农产品优势区。做大做强牧同牛奶等本土品牌。二是建设有机旱作农业样板区。加强与中国农科院、省农科院、山西农大等院校合作,依托特色小杂粮,重点开发功能食品和药食同源产品。发挥大同杂粮创新中心引领作用,实施地力提升、旱作良种、旱作技术、农业机械推广4项计划,总结推广秋膜覆盖试点经验,有机旱作农业面积达到4.5万亩。力争创建灵丘、天镇、广灵3个省级示范县,引领形成具有大同特色的有机旱作农业技术路径,产出“大同好粮”。三是建设特色农业观光区。在大张高铁123公里沿线规划布局10万亩特色农业观光区,引导沿线19个乡镇68个行政村调整种植结构,集中连片发展杏果、黄花、中药材、苜蓿、蔬菜等经济作物,打造经济价值高、生态景观美、综合效益优的农业产业基地,形成地域特色浓厚的调产区、风景区和农民增收区,力争年底前阳高杏韵小镇全国农村产业示范园等一批大项目好项目集中建成和投产。四是建设美丽宜居乡村示范区。重点打造天镇贾家屯、大同吉家庄、灵丘红石塄、广灵涧西村等一批宜居宜业示范乡村,有效破解自来水、卫生间、宽带、管网、电视户户通、市县有线电视节目接入“六入户”等瓶颈,建立健全农村生活垃圾收运处置体系,规划建设农村污水处理设施,推进农村“厕所革命”,让乡村美起来。同时,建成“四好农村路”1528公里,实施267户农村地质灾害治理搬迁工程,改善农村的生产生活条件。五是发展壮大县域经济。县区长主抓10个带动性强的好项目,认证5个“三品一标”特色农产品,建设3万亩农业标准化基地,推动县域经济提质增效。市里拿出1亿元资金扶持县域经济和民营经济发展,奖励当年引进签约、当年建成投产的转型项目。针对规上企业少、弱、小的现状,通过政策扶持、资金奖励、技术攻关等手段,加快小升规步伐,力争各县规上企业数量都要实现一年翻番,以县域经济的大发展强化乡村振兴的硬支撑。

(六)文旅振兴行动

文化旅游产业是具有乘数效应的朝阳产业,文化是城市综合竞争的灵魂,文化强则城市兴,大同是文化璀璨的城市,文化旅游产业是大同资源型经济转型的主攻方向。要坚持把文旅产业作为战略性支柱产业来抓,瞄准康养游、长城游、乡村游,丰富全域旅游业态,提升文旅产业综合效益,确保旅游总收入、接待国内外游客总数同比增长16%。重点做好五个方面工作。一是创建全域旅游示范区。编制全域旅游发展规划,出台《促进旅游投资和消费的意见》,与省旅发委签订旅游工作支持协议,提速国家级旅游业改革创新先行区建设。深化云冈、恒山、古城等景区景点管理体制机制改革,加快白登山万龙滑雪场项目建设,创建浑源、灵丘、大同县3个国家全域旅游示范区,打造各具特色的文化旅游新名片,以点带面,引领全域旅游发展。二是建好长城旅游一号公路。编制长城旅游开发专项规划,重点打造东起天镇李二口、西至左云摩天岭249公里的国家一号风景大道。建成后,将形成横贯天镇、阳高、新荣、南郊、左云5个县区的绿色廊道、旅游通道、风光大道,成为生态旅游的观光路、助农增收的致富路。三是发展乡村旅游。优先开发桑干河流域、神溪湿地、唐河流域、火山群等旅游资源富集区。培育上下北泉、车河有机农业社区等一批新型乡村旅游示范点。推进阳高农业生态观光与文化旅游综合体建设。加快云冈小镇、恒山古镇、新平堡小镇等10个特色小镇建设,为全市农村城镇化创造示范。四是建设综合康养产业区。依托国发〔2017〕42号文件政策支持,积极引进复华置地、卓越集团、华体集团、中能建投等战略投资者,加快综合康养产业区建设,构建养老、养生、医疗、文化、体育、旅游等诸多业态相融合的新型健康产业发展模式。重点扶持阳高康养休闲养生基地建设等项目,打造国家级康养基地。五是加快文旅项目建设。加快推进文旅新业态、基础建设、康养度假、乡村旅游开发、旅游小镇、特色旅游、文化演艺等7个方面34个文旅融合项目建设,着力补齐短板,丰富文旅业态,吸引更多国内外游客。

(七)招商引智行动

招商引智是实施创新驱动、加快转型升级的重要支撑。开展招商引智行动,重中之重是聚焦高科技工业和新兴工业,引进科技含量高、带动能力强的转型项目,引进国际尖端人才和产业领军人物。今年要突出抓好三项工作。一是紧扣“招什么”,把握国家政策导向和市场资金投向,围绕信息技术、高端装备制造、新能源汽车等九大新兴产业,精心编制招商方案,谋划优质转型项目,进行系统化、定点式、有序性的精准招商和专业招商。落实凤凰人才计划,实施千名博士百名院士引进工程。开展与清华启迪、北大燕园、中科院全科盟深入合作,实现1000名科技人才服务大同。二是紧扣“怎样招”,举办首届新能源大同高峰论坛、京津冀走进大同发展论坛、世界500强企业对话大同等活动,把适合大同发展的产业和项目引进来,年内力争引进东部地区一批重大项目,实现百家央企名企走进大同。定期召开企业家座谈会,建立企业家联络联系机制,鼓励同商同才回家创业,实行以商招商、产业链招商。三是紧扣“靠什么招”,把开发区作为招商引智的主战场,重兵重器支持开发区建设,年底建成100万平方米标准化厂房,为年内谋划100个新项目落地创造条件。加快金控集团和产业转型项目投资管理公司运营,设立100亿元转型发展基金,解决企业融资难、融资贵的问题,为项目落地扫清障碍。着眼于解决引进人才的生活问题,提供具备拎包入住条件住房2000套,解决引进人才配偶、子女的工作和上学问题。

我们要牢固树立“招商引资就是推动转型、服务项目就是促进转型”的工作理念,千军万马跑项目,千辛万苦攻项目,千方百计上项目,要始终坚持以项目统领发展,以项目论英雄,用项目检验作风,用项目检验能力。要发挥主观能动性,主动“走出去、请进来”,坚决盯住项目、盯住人才、盯住成果,把招商引智作为增动能、补短板的重点工程,变成解决结构性矛盾和产业单一的突破口。

(八)创新培育行动

创新是引领发展的第一动力,是建设现代化经济体系的战略支撑。实施创新培育行动,核心是促进大同大众创业、万众创新上水平。今年重点抓好四项工作。一是搭建创新平台。吸引行业领军人物和院士来同建立国家实验室、新型研发机构、产业研究院等各类创新平台,促进产学研深度融合。加快同药集团生物制药院士工作站建设,引领更多企业开发和购买科技创新成果。加强专业化众创空间和科技企业孵化器建设,新培育省级双创示范基地2家,努力创建国家级双创示范基地。二是培育创新主体。重点依托70研究所提升高端装备制造业突破能力,发挥大齿集团国家级国际科技合作基地的引领作用,推动同煤、同药、山柴等更多企业实施重大科技项目,提升科技研发和创新能力。鼓励企业设立研发机构,力争新培育市级企业技术中心4户、省级企业技术中心2户。支持非公经济创新发展,鼓励新成新材料等民营企业加大新能源材料装备技术研究与开发。今年力争创办小微企业2000户以上,培育省级“专精特新”中小企业10户以上。三是储备创新人才。充分发挥大同大学人才资源优势,落实与省教育厅共建大同大学战略合作,加快建设大数据、能源产业、通用航空和对外经贸学院,培养地方适用型、市场急需型人才。整合全市职业教育资源,优化学校布局和专业设置,创新现代学徒制等灵活办学模式,培养更多的技能型人才。四是落实创新政策。充分利用国家级、省级重点实验室、工程研究中心、科技基础条件平台及高等院校、科研院所等资源,今年市政府将制定出台“创新产业券”激励政策,分10万、20万、30万、50万、100万五个等级为创新主体发放科技创业券,凡是来自进行创新的主体,办理注册时即可拿到创新券和免费提供的办公场所,支持科技型中小微企业开展研发活动和科技创新。各级各部门要营造尊重创造、鼓励创新、支持创业的文化氛围,凝聚创新创业热情和力量,跑出大同创新“加速度”。

(九)脱贫攻坚决胜行动

打好脱贫攻坚战是推动城乡融合发展、决胜全面建成小康社会的现实选择。今年,全市265个贫困村退出、10万贫困人口脱贫,阳高、大同、灵丘三个贫困县脱贫摘帽,左云县贫困村、贫困人口全部退出。这是硬任务、硬要求,必须下足绣花功夫,持续精准施策,抓好六项工作。一是强化产业扶贫。每个贫困县扶持一批特色种植养殖项目、建设一个农产品加工厂、新建一个扶贫产业园区、包装一个旅游扶贫项目,每个贫困村建一个光伏电站,发展壮大一批贫困村集体经济,力保所有贫困村集体经济收入超过5万元,力争一半以上的贫困村集体经济收入突破10万元。确保每个贫困村要有带动农民增收致富主导优势产业,确保每一户农民有稳定持续多元的收入渠道。二是加快易地搬迁。进一步强化部门协同作战能力,超常规审批,超常规运作,确保今明两年完成6.83万人易地扶贫搬迁任务,摘帽三县易地扶贫搬迁两年任务一年完。三是促进就业扶贫。放宽贫困群众就业条件,拓宽劳务输出对接机制,增加贫困群众就业机会,贫困劳动力技能培训2万人,安置就业2万人。造林合作社吸收贫困人口数量要占总人数60%以上,人均劳务收入达到1.2万元以上,同时继续扩大聘用建档立卡贫困人员为生态护林员的数量,增加贫困人口工资性收入。四是助推教育扶贫。落实国家教育资助五项政策,健全完善覆盖学前教育、义务教育、普通高中教育、中等职业教育和高等教育资助体系,推动建立从学前教育到小学、中学、大学直至就业的“一条龙”帮困包扶机制,实现贫困村九年义务教育阶段无因贫辍学的学生。五是加强兜底扶贫。将符合条件的低保户、五保户和残疾人全部纳入建档立卡贫困人口。将完全或部分丧失劳动能力的贫困人口全部纳入农村低保。全面落实“三保险、三救助”和“136”健康扶贫政策,解决因病致贫、因病返贫问题。六是实施整村提升。全市选出60个村作为示范村,今年退出的贫困村占比不少于三分之二,实施村容村貌和户容户貌提升工程,4月初召开动员会,7月底召开现场观摩会。3个摘帽县在示范村基础上实现全覆盖,其他县区覆盖率达到50%以上。实现村内巷道硬化、入户道路硬化、农户庭院硬化,村容村貌、户容户貌明显改善,水、电、路、网、垃圾处理等基础设施配套,贫困群众生产生活条件得到有效改善。

(十)民生普惠行动

解决民生问题是为政的根本,改善民生状况是最大的政绩。我们要从细处实处入手,为老百姓办好“关键小事”、办成暖心实事,交出合格答卷。今年要着力抓好五项工作。一是抓好重点民生事业。教育方面,实施第三期学前三年行动计划,解决好“入公办园难”、“入民办园贵”的问题。启动“县管校聘”改革,增强学校办学活力。完成“全面改薄”任务,促进城乡义务教育均衡发展。坚决打赢高中教育翻身仗,办好每一所学校,荣耀每一位教师,幸福每一个家庭!就业创业方面,实施就业帮扶和质量提升工程,抓好高校毕业生、“零就业”家庭等重点群体的就业再就业工作。医疗卫生方面,健全“三医联动”长效机制,加快推进医联体建设,深化公立医院改革,传承发展中医药事业,启动国家级医养结合试点工作,扩大跨省异地就医直接结算范围。社会保障方面,实施全民参保登记,强化养老、医疗、社会救助等兜底作用,为优抚、五保、高龄和贫困老人办理意外伤害保险,织密扎牢民生保障网。二是大力发展文体事业。实施文化惠民工程,建设“书香大同”。御东图书馆投入运营。承办好中国第二届青年运动会。落实好山西航空职业技术学院项目。开展丰富多彩的市民健身运动,让老百姓的身体更健康,心情更舒畅!三是强化住房多元供给。全面加强房地产市场监管,依法规范售房,保持房价走势平稳。改造城市消极空间,盘活古城片区,梯次开发御东新区。加大保障房建设和政府回购安置力度,启动限价房建设。实施租售并举,培育房屋租赁市场,适当增加租赁住房用地供应,有序解决租房问题。解决好县区房地产去库存,盘活一批呆滞存量房。四是加强生态文明建设。落实大气十条、水十条、土十条,逐步扩大禁煤区,提速“煤改电”“煤改气”,着力提升“大同蓝”。落实河长制,实施桑干河等重点流域和城镇生活污水处理设施扩容提标改造工程,着力建设“大同清”。加快永定河综合治理、采煤沉陷区地质环境专项治理,完成营造林19.57万亩,厚植“大同绿”,全面打赢污染防治攻坚战,真正实现绿水青山就是金山银山。五是提升安全维稳水平。落实“四铁”要求,压实安全生产责任,强化安全管理,突出抓好煤矿、非煤矿山、道路交通、食品药品安全等重点行业领域专项整治,推动安全生产形势持续好转。完善立体化治安防控体系,打赢扫黑除恶专项斗争,营造安定祥和的社会环境。有效调处扶贫、国企改革、环保、征地拆迁等重点领域的矛盾纠纷,保障群众合法利益诉求,不断提升“幸福大同”的品质内涵。

同时,要继续加强国防教育和国防动员建设,持续开展双拥工作,推进军民深度融合发展。发展档案事业,实现市县方志年鉴全覆盖。支持工会、共青团、妇联等人民团体更好地发挥桥梁纽带作用。做好外事、侨务、港澳、对台工作。加强民族宗教、对外援疆等工作。

在做好以上各项工作的同时,市政府还要认真办好10件惠民实事。1.完成公园游园20公里健身步道建设。2.启动“光明扶贫工程”,为全市所有建档立卡贫困人口提供白内障免费救治服务。3.实施市内25个停车场免费开放。4.新建10座过街天桥。5.进一步扩大残疾预防重点干预和残疾儿童抢救性康复项目实施规模。6.在6个国定贫困县免费“两癌”检查服务由建档立卡农村妇女扩大到全部农村妇女。7.继续为怀孕妇女提供免费产前筛查与诊断服务。8.新建30个农村老年人日间照料中心。9.完成免费送“千场大戏、万场电影”文化惠民任务。10.实施全民技能提升工程,全年培训5万人以上。要树立人民情怀,把每一件事情办到老百姓的心坎上。

三、全面加强政府自身建设

重任千钧惟担当,打铁还需自身硬。面对改革发展的艰巨任务,我们将以政治建设为统领,以服务型政府建设为根本,全面提升政府自身建设水平,推动“两个持久”不断向纵深发展,在全面决胜小康的关键期,走好新征程,探出新路子,创造新业绩。

紧跟核心,把牢方向。深入推进“两学一做”学习教育常态化制度化,扎实开展“不忘初心、牢记使命”主题教育,牢固树立“四个意识”,切实增强“四个自信”,坚决做到“四个服从”,(下转第三版)(上接第二版)坚决拥护习近平总书记这个党的核心、军队统帅和人民领袖,始终坚持以习近平同志为核心的党中央权威和集中统一领导,在政治立场、政治方向、政治原则、政治道路上始终同以习近平同志为核心的党中央保持高度一致。

尊崇法治,从严治政。实施全面依法治国基本方略,落实《宪法修正案》,把政府活动全面纳入法治轨道。严格执行人大及其常委会的决议决定,依法接受人大法律监督,自觉接受政协民主监督,主动接受社会监督和舆论监督。坚持科学决策、民主决策、依法决策,广泛听取各方意见包括批评意见。主动接受《监察法》监督,实现对所有行使公权力的公职人员监察全覆盖。加强审计监督,健全完善政府工作内审制度,加大工作力度,扎紧制度笼子。推进政府机构改革,优化政府机构设置和职能配置,形成职责明确、依法行政的政府治理体系。

干字当头,狠抓落实。以拼搏为美,向行动致敬,自觉践行省委“五倡导五反对”,珍惜组织给的平台,感恩人民赋予的责任,用行动诠释忠诚,用责任书写担当。始终坚持激情干事。起步就是冲刺、开局就是决战,以钉钉子的精神,从初一抓到年三十,保持“五加二、白加黑、雨加雪”的状态,多为发展想办法,少为困难找理由,撸起袖子加油干,样样工作争一流!始终坚持精准干事。把握政府工作的重点、难点、堵点,找准不平衡、不充分发展的问题、短板、瓶颈,用心用情用力,研究针对性举措,制定有效性对策,细化任务书,列出时间表,绘出路线图,确保每一项工作挂图作战、跑表计时、到点验收!始终坚持开拓干事。“山再高,往上攀,总能登顶;路再长,走下去,定能到达。”逢山敢开路,遇水能架桥,立下愚公志,敢啃硬骨头,积极投身转型升级的大同实践,做到胸中有法、手上有招、脚下有路,确保全年任务圆满完成!

优化环境,提升效能。与中央要求对表,与发达地区对标,与国际通行惯例对接,完善形成法治化、国际化、便利化的发展环境。积极转变政府职能,深化“放管服效”改革,落实企业投资项目承诺制,打造“六最”营商环境。坚决清理群众和企业办事的各类证明,凡是没有法律法规依据的一律取消,让老百姓办事“只进一扇门、最多跑一次”。牢固确立“时间就是金钱,效率就是生命”的工作理念,坚持说了就干、马上就办,限时办结、超时问责,凡在同落地项目,1天之内必须完成注册和税务登记等手续,真办、快办、帮着办,以真情和诚信赢得企业点赞!

遵规守纪,廉洁奉公。坚决把纪律和规矩挺在前面,全面加强党风廉政建设,保持良好政治生态。构建亲清新型政商关系,形成感情共融、合作共建、成果共享的生动局面。落实中央八项规定,驰而不息纠正“四风”,力戒形式主义、官僚主义。严肃财经纪律,节约财政开支,最大限度降低行政运行成本。坚持过紧日子,把有限的钱用到民生最需要的地方。政府工作人员要廉洁修身,勤勉尽责,干干净净为人民做事,决不辜负人民公仆的称号。

各位代表,大同具有开改革风气之先的深厚历史底蕴,已经积蓄了砥励前行的强大动力,当前正处在一个大有可为的历史机遇期。置身日新月异的新时代,面对全市340万人民的殷切期盼,让我们高举习近平新时代中国特色社会主义思想旗帜,在省委、省政府和市委的坚强领导下,不忘初心,牢记使命,为决胜全面建成小康社会、为加快推进现代化建设、为早日实现美丽富裕幸福大同作出我们新的更大的贡献!
